# A novel lipid droplet-related prognostic signature reveals CIDEC as a key biomarker for pancreatic adenocarcinoma

**DOI:** 10.3389/fonc.2026.1845259

**Published:** 2026-07-28

**Authors:** Xuyang Shao, Jun Zhu, Xiangyu Gao, Siyu Li, Fei Zhong, Yanlong Shi

**Affiliations:** 1 Department of General Surgery, Guoyang County People’s Hospital, Guoyang, Anhui, China; 2 Department of Oncology, Guoyang County People’s Hospital, Guoyang, Anhui, China; 3 Department of Oncology, The Fifth Affiliated Hospital of Anhui Medical University, Fuyang, Anhui, China; 4 Department of Oncology, Linquan County People’s Hospital, Linquan, Anhui, China; 5 Department of Oncology, The First Affiliated Hospital of Anhui Medical University, Hefei, Anhui, China; 6 Department of General Surgery, The Fifth Affiliated Hospital of Anhui Medical University, Fuyang, Anhui, China

**Keywords:** CIDEC, competing endogenous RNA, lipid droplet-related genes, pancreatic adenocarcinoma, prognostic signature

## Abstract

**Purpose:**

Reprogramming of lipid metabolism is acknowledged as a key characteristic of cancer. This research aimed to examine the potential of lipid droplet-associated genes (LDRGs) in forecasting patient outcomes and the effectiveness of immunotherapy in pancreatic adenocarcinoma (PAAD).

**Methods:**

A predictive signature was developed through LASSO Cox regression analysis. Survival predictions were assessed via Kaplan-Meier survival analysis, along with univariate and multivariate Cox regression analyses. Associations between LDRGs and tumor-immune infiltration, as well as immune checkpoints, were examined using Spearman’s analysis. The pivotal gene CIDEC within the LDRGs was pinpointed, and a CIDEC-associated competing endogenous RNA (ceRNA) regulatory network was established employing comprehensive bioinformatics analysis. The pro‐tumor phenotype of CIDEC was explored in PAAD cell lines through molecular experiments.

**Results:**

A signature model related to LDRGs for determining the prognosis of PAAD was constructed, based on which patients were classified into two risk groups. A nomogram accompanied by calibration curves was constructed, which proved a robust predictive value. Additional investigations revealed that these prognostic LDRGs are significantly associated with levels of immune infiltration and the expression of immune checkpoints. Through ceRNA network analysis, a regulatory axis involving LINC00365, hsa-miR-32-5p, hsa-miR-363-3p, and CIDEC was identified in PAAD. Experimental evidence indicates that elevated CIDEC expression markedly enhances proliferation, migration, and invasion capabilities in PAAD cell lines while simultaneously suppressing apoptosis. Conversely, reduced CIDEC levels produce the opposite effects.

**Conclusion:**

In conclusion, our novel prognostic signature model has promising applications in PAAD patients. Within this framework, CIDEC may serve as a valuable candidate biomarker and a potential target for therapeutic intervention, thereby providing a basis for future research. The LINC00365/hsa-miR-32-5p/hsa-miR-363-3p/CIDEC regulatory axis may be involved in the progression of PAAD.

## Introduction

Pancreatic adenocarcinoma (PAAD) is associated with a substantial global mortality burden, exhibiting a five-year overall survival (OS) rate as low as 12%, which underscores the considerable difficulties in its management (1). Its early manifestations are atypical, most cases are identified at the middle or advanced stages with a grim prognosis (2). Although PAAD does not rank among the top of all malignant tumors in terms of incidence, its mortality ranking persists at a relatively elevated level (3). Diagnostic and therapeutic advances such as neoadjuvant therapy, genetic testing, and molecular typing have resulted in longer survival for some patients, but their overall prognosis is still not optimistic (4, 5). Therefore, confronting the grim prognosis of PAAD patients, it is imperative to filter out effective prognostic signatures and new prognostic markers.

Lipid droplets (LDs) are specialized organelles that store lipids and play essential roles in diverse cellular processes, including gene expression regulation and metabolic pathways (6). Lipid metabolism abnormalities are manifested in numerous pathological states, including obesity, insulin resistance, inflammatory responses, neuronal diseases, and cancer (7–9). LDs homeostasis impairment is a significant pathogenic factor that may be closely linked to the pathogenesis of various human and animal tumors, as well as their associated cachexia (10). Several studies have also demonstrated that impaired LDs homeostasis significantly impacts the prognosis of malignant tumors, including gastric cancer (11), colorectal cancer (12), and hepatocellular carcinoma (13). Furthermore, the accumulation of abnormal LDs is closely associated with enhanced tumor cell proliferation, resistance to chemotherapy and radiation therapy, and a more aggressive phenotype in PAAD (14). Importantly, there is a close interaction between lipid droplet metabolism and the tumor immune microenvironment (TIME). For example, lipid-laden cancer cells can suppress the function of effector T cells and promote the infiltration of cells that suppress immune responses, including tumor-associated macrophages (TAMs) and myeloid-derived suppressor cells (MDSCs) (15). It has been shown that the reprogramming of lipid metabolism within the TIME microenvironment may influence the expression of immune checkpoints and the efficacy of immunotherapy (16). However, the precise mechanisms by which lipid droplet-related genes (LDRGs) regulate the progression of PAAD and the remodeling of the immune landscape remain largely unexplored.

Accumulating evidence has revealed the critical role of lncRNA-miRNA-mRNA competitive endogenous RNA (ceRNA) in cancer progression and metastasis (17). One study has demonstrated that the ceRNA mechanism could provide a reference standard for molecular biological research on PAAD in the future (18). However, studies focusing on the comprehensive value of LDRGs in PAAD progression remain scarce, particularly those involving the molecular mechanisms of ceRNA regulatory networks in PAAD progression.

This research constructed a new prognostic model founded on LDRGs. Furthermore, the function of the key gene CIDEC in PAAD cells was validated through *in vitro* experiments, and a potential CIDEC-related ceRNA regulatory axis was identified. The objective of our work is to assess the clinical outcomes for PAAD patients by employing a lipid droplet-associated prognostic model and to clarify its potential implications for targeted therapeutic approaches.

## Materials and methods

### Clinical specimen collection and data acquisition

Paraffin-embedded tissue sections of clinical samples, comprising both PAAD tissues and matched normal pancreatic tissues from adjacent areas, were supplied by the Pathology Department at The Fifth Affiliated Hospital of Anhui Medical University. The research adhered to the principles outlined in the Declaration of Helsinki and received approval from the Ethics Committee of The Fifth Affiliated Hospital of Anhui Medical University (KYPJ-2026-054). We retrieved the RNA expression profiles and clinical-pathological information for PAAD from The Cancer Genome Atlas (TCGA) (https://portal.gdc.cancer.gov/) database, and performed data preprocessing using R software (version 4.0.3). **Table 1** presents the clinical details of PAAD patients. Using the search term “lipid droplet-associated genes” and filtering for “relevance score >20”, a total of 26 LDRGs (**Supplementary Table 1**) from the GeneCards (https://www.genecards.org/) database were identified.

**Table 1:** The clinical information of PAAD patients in TCGA.

Characteristic	Subgroup / Value	Number
Status	Alive	86
	Dead	93
Age	Mean (SD)	64.6 (10.9)
	Median [MIN, MAX]	65 [35,88]
Gender	FEMALE	80
	MALE	99
pT stage	T1	7
	T2	24
	T3	143
	T4	3
	TX	1
pN stage	N0	50
	N1	120
	N1b	4
	NX	4
pM stage	M0	80
	M1	5
	MX	94
pTNM stage	I	21
	II	147
	III	3
	IV	5
Grade	G1	31
	G2	96
	G3	48
	G4	2
	GX	2

### Differential expression, correlation, and functional enrichment analyses of LDRGs

Using the “limma” and “reshape2” software packages, we analyzed the differential expression of 26 LDRGs in PAAD and normal tissues. The protein-protein interactions (PPI) between 26 LDRGs were generated from the STRING (https://string-db.org/) database. Then, the LDRGs were analyzed using the “clusterProfiler” package for Gene Ontology (GO) and Kyoto Encyclopedia of Genes and Genomes (KEGG) analyses. Bubble plots were employed to visualize the enrichment results.

### LDRGs-related prognostic signature analysis

We used univariate Cox regression analysis to identify LDRGs with prognostic value. A LDRGs-related prognostic signature was then constructed using LASSO Cox regression analysis. After centralized and standardized calculations, the risk scores are as follows: Riskscore = (0.0667)*CIDEC + (0.1043)*HILPDA + (-0.4689)*LIPE + (0.2205)*MYC + (0.0444)*PNPLA3 + (0.1312)*PPARG. PAAD patients were divided into two groups (low-risk and high-risk), and survival curves for both groups were plotted using the Kaplan-Meier method. To evaluate the validity of the signature, the receiver operating characteristic (ROC) curve was introduced. The “Survival” package, “survminer” package and “timeROC” package in R language were employed. Moreover, the prediction of overall survival at 1, 2, and 3 years was performed using a nomogram. Calibration curves and the C-index were used to evaluate the consistency of the nomogram.

### Immune-related analysis

The “Gene” and “Scna” modules of TIMER (https://cistrome.shinyapps.io/timer/) could visualize the relationship between LDRGs expression levels and the degree of immune infiltration. Spearman method was conducted to calculate the relationship between LDRGs expression and immune checkpoint-related genes.

### CeRNA network construction

CIDEC-related miRNA targets were identified through the starbase (https://starbase.sysu.edu.cn/). We then explored lncRNA targets interacting with miRNAs with the help of miRNet (https://www.miRNet.ca/). Furthermore, the expression levels and prognostic significance of these miRNA and lncRNA targets in PAAD were examined using starbase and Kaplan-Meier plotter.

### Cell culture and transfection

The human pancreatic ductal epithelial cell (HPDE6-C7) and PAAD cell lines (BxPC-3, CFPAC-1, MIA PaCa-2, PANC-1, PATu8988T) were purchased from Procell Biotech (Wuhan, China). Cells were cultivated in DMEM (Gibco, catalog number C11965500BT) containing 10% fetal bovine serum (Servicebio, catalog number WGG8001) and 1% penicillin and streptomycin (Servicebio, catalog number G4003) in 5% CO2 at 37 °C, respectively. The CIDEC-overexpressing lentiviruses (CMV-MCS-3flag-EF1-ZsGreen-T2A-PURO) and short hairpin RNA (shRNA)-encoding lentiviruses (U6-MCS-CMV-ZsGreen-PGK-PURO) were acquired from GeneChem Co. Ltd. (Shanghai, China). The shRNA target sequences are as follows: ATAAGCCTGCCAAGAAGATTG, TGAACCCACAGGACTTCATTG, and TCCCGACCTGTCTGAAGATAC. Transfection should be performed according to the instructions when the cell confluence reaches approximately 50%.

### Immunofluorescence

After paraffin-embedded tissue sections were deparaffinized and rehydrated, antigen retrieval was performed in citrate buffer (10 mM, pH 6.0). Overnight incubate the tissue sections with anti-CIDEC primary antibody (1:100) at 4 °C after blocking with 5% normal goat serum, then incubate with fluorescently labeled secondary antibody for 1 hour at room temperature. Perform counterstaining of the cell nuclei using DAPI. Images were captured using a fluorescence microscope.

### Western blotting

The CIDEC protein was isolated from cells using RIPA buffer (Beyotime, catalog number P0013B), and then the concentration of CIDEC protein was measured by the BCA method. The proteins were separated by SDS-PAGE (10% gel) and subsequently electroblotted onto a PVDF membrane (Servicebio, catalog number G6044). The membrane was sealed with skim milk (Servicebio, catalog number GC310001), then incubated overnight at 4 °C in CIDEC antibody (Proteintech, catalog number 12287-1-AP), followed by a 1-hour incubation with the secondary antibody (Servicebio, catalog number GB23303) at room temperature. Finally, visualization was achieved through enhanced chemiluminescence.

### RNA extraction and qPCR

In summary, RNA extraction was carried out with TRIzol reagent (Invitrogen, catalog number 12183–555). To generate cDNA from the isolated RNA, the PrimeScript ™ RT Master Mix kit (Takara, catalog number RR036A) was employed. QPCR analysis was conducted using TB Green^®^ Premix Ex Taq™ II (Takara, catalog number RR820A). The expression of CIDEC was normalized to the internal reference gene GAPDH. The primer sequences utilized were as follows: for CIDEC, the forward primer was AAGTCCCTTAGCCTTCTCTACC and the reverse primer was CCTTCCTCACGCTTCGATCC; for GAPDH, the forward primer was GGAGCGAGATCCCTCCAAAAT and the reverse primer was GGCTGTTGTCATACTTCTCATGG.

### CCK-8 assay

For the CCK-8 assay, cells were seeded at a density of 3 × 10³ cells per well in a 96-well plate. Following the kit instructions, the cells were incubated with the CCK-8 solution for durations of 24, 48, 72, 96, and 120 hours. Absorbance at 450 nm (optical density) was then recorded using a microplate reader (Tecan, Switzerland).

### Wound-healing assay

Cells (3 × 105 cells per well) that had been engineered to overexpress or have reduced levels of CIDEC were seeded into 12-well plates. Once the cells achieved a confluency of 90% or higher, a sterile pipette tip was used to create a linear scratch wound in the middle of the adherent cell layer. Following wound induction, the cells were maintained in a medium lacking serum. Photographs of the wound area were taken with an optical microscope (Olympus, Japan) at the initial time point (0 hours), as well as after 24 and 48 hours.

### Transwell assay

We suspended CIDEC-overexpressing or CIDEC-knockdown cells in serum-free medium and then added them to the upper well of the Transwell chamber (LABSELECT, catalog number 12100089). These chambers were pre-coated with Matrix Gel (Beyotime, catalog number C0372) and placed in a 24-well plate. The lower chamber was filled with enriched medium (10% FBS), which serves as a chemical inducer. Cells that had traversed the cell membrane were fixed with 4% paraformaldehyde (Beyotime, catalog number P0099) and stained with 0.1% crystal violet (Servicebio, catalog number GC307002) after incubation at 37 °C for 24 hours. The resulting stained samples were examined using a light microscope (Olympus, Japan).

### Flow cytometry

Cells with CIDEC overexpression or knockdown were digested with 0.25% trypsin, followed by resuspension. The Annexin V-FITC apoptosis detection kit (Beyotime, catalog number C1062L) was utilized for cell apoptosis detection. Apoptosis rate measurement was performed using flow cytometry (BD Biosciences, USA).

### Statistical analysis

R 4.1.3 and Prism 8 software were employed to perform statistical analyses. Significant differences between variables were assessed using the chi-square test and Student’s t-test. Survival disparities among different groups were assessed by Kaplan-Meier survival curves combined with log-rank tests. *P* < 0.05 was denoted statistically significant.

## Results

### Expression of LDRGs in PAAD

**Figure 1:**
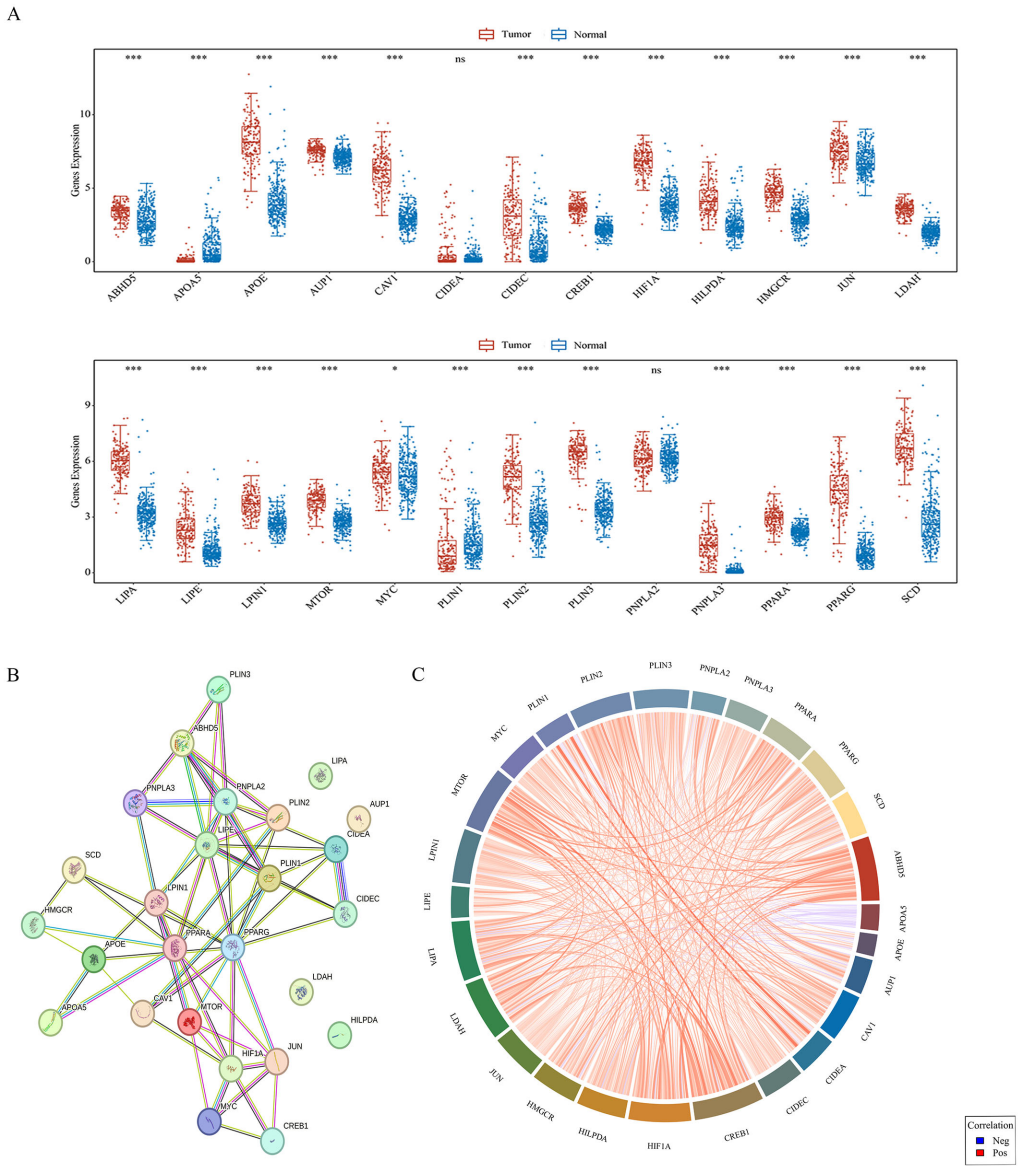
The distinct expression patterns of LDRGs in PAAD. **(A)** Expression differences of 26 identified LDRGs between PAAD tumor tissue (shown in red) and adjacent normal tissue (shown in blue). **(B)** The PPI network demonstrating the interactions among these LDRGs. **(C)** Correlation analysis of 26 LDRGs. *P < 0.05, ***P < 0.001.

A comparative analysis of 26 LDRGs in PAAD, utilizing data from the TCGA and GTEx databases, revealed 24 genes with significant differential expression (**Figure 1A**). The expression levels of 22 genes (ABHD5, APOE, AUP1, CAV1, CIDEC, CREB1, HIF1A, HILPDA, HMGCR, JUN, LDAH, LIPA, LIPE, LPIN1, MTOR, MYC, PLIN2, PLIN3, PNPLA3, PPARA, PPARG, and SCD) in PAAD were upregulated relative to those in the pancreatic tissues of normal subjects. Conversely, the expression of two genes, APOA5 and PLIN1, was significantly reduced (**Figure 1A**, all *P* < 0.05). Construction of a PPI network via the STRING database identified ABHD5, HIF1A, PNPLA2, LIPE, PLIN1, LPIN1, PPARA, and PPARG as central hub genes (**Figure 1B**). Furthermore, we performed a correlation analysis based on the expression levels of LDRGs, which showed a general positive correlation in PAAD (**Figure 1C**).

### Functional enrichment analysis of LDRGs in PAAD

**Figure 2:**
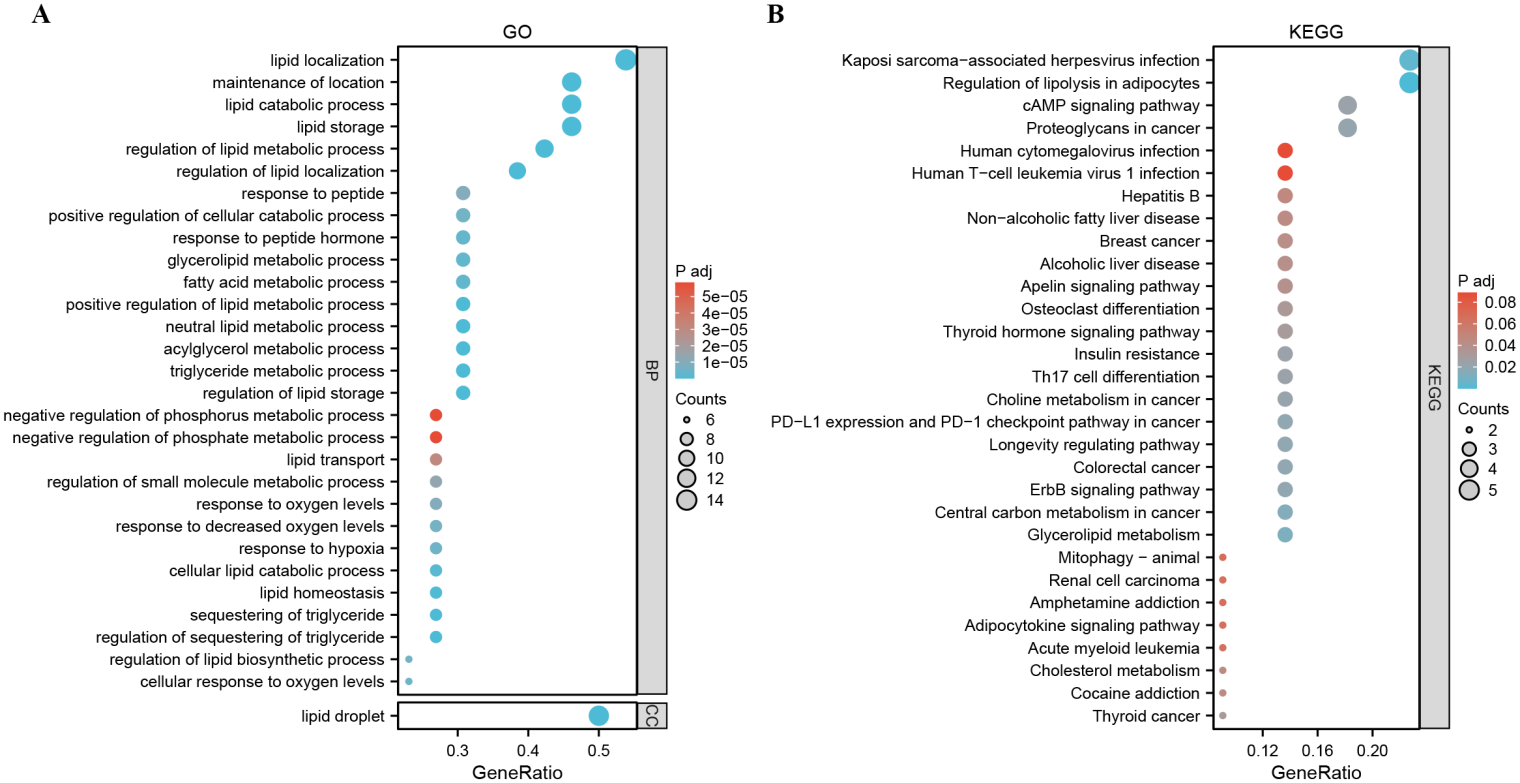
Functional enrichment analysis. GO **(A)** and KEGG **(B)** pathway enrichment analysis of the 26 LDRGs.

To gain deeper insights into the biological roles of the differentially expressed LDRGs, GO and KEGG pathway enrichment analyses were conducted (**Figures 2A, B**). GO biological process (BP) analysis revealed that 26 LDRGs were mainly associated with lipid localization, maintenance of location, lipid catabolic process, lipid storage, and regulation of lipid metabolic process. Regarding cellular components (CC), the analysis indicated a strong association of these genes with lipid droplets. We also found that these 26 LDRGs were mainly enriched in kaposi sarcoma−associated herpesvirus infection, regulation of lipolysis in adipocytes, cAMP signaling pathway, and proteoglycans in cancer in KEGG pathway analysis.

### Construction of the prognostic LDRGs model in PAAD

**Figure 3:**
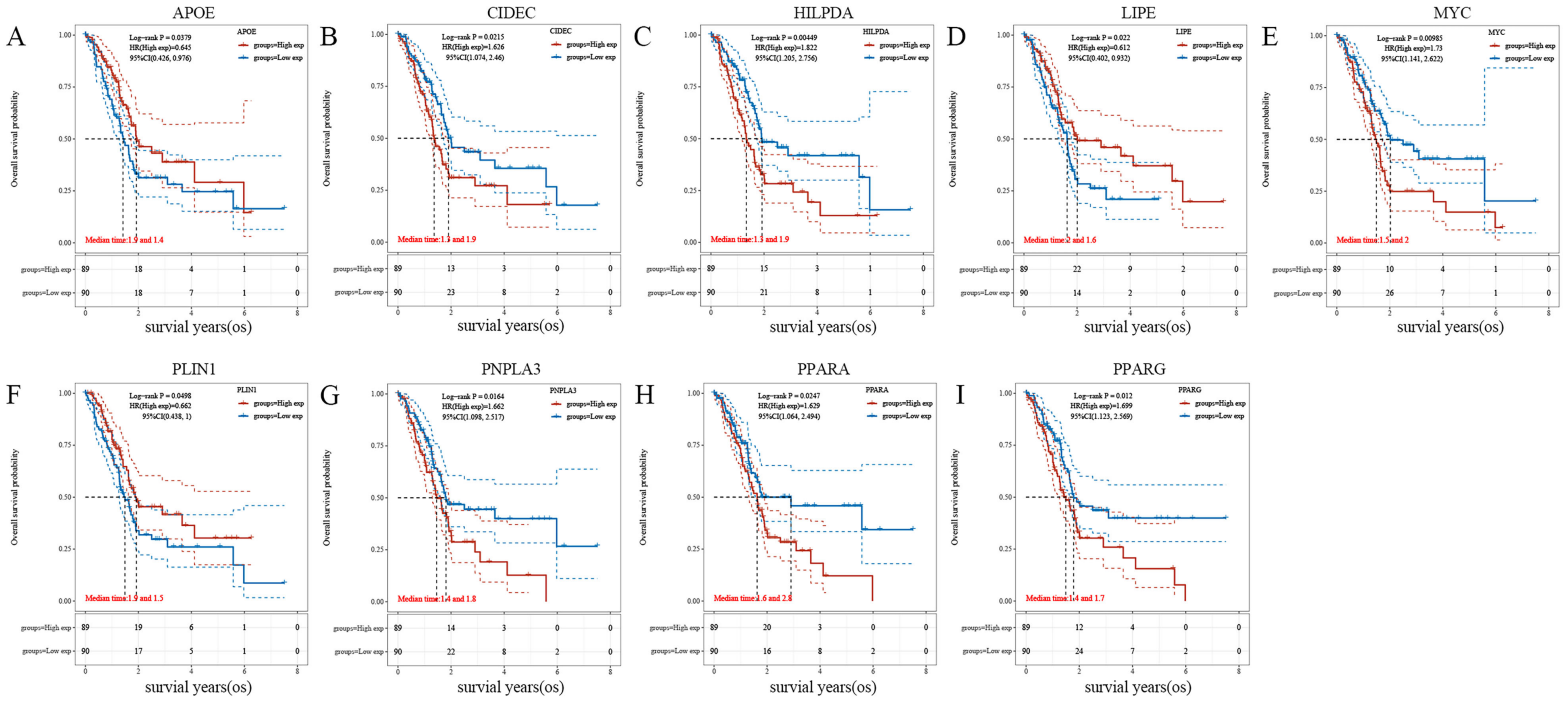
The prognostic value of LDRGs for PAAD patients in the high-/low-expression groups. **(A)** APOE **(B)** CIDEC **(C)** HILPDA **(D)** LIPE **(E)** MYC **(F)** PLIN1 **(G)** PNPLA3 **(H)** PPARA **(I)** PPARG.

**Figure 4:**
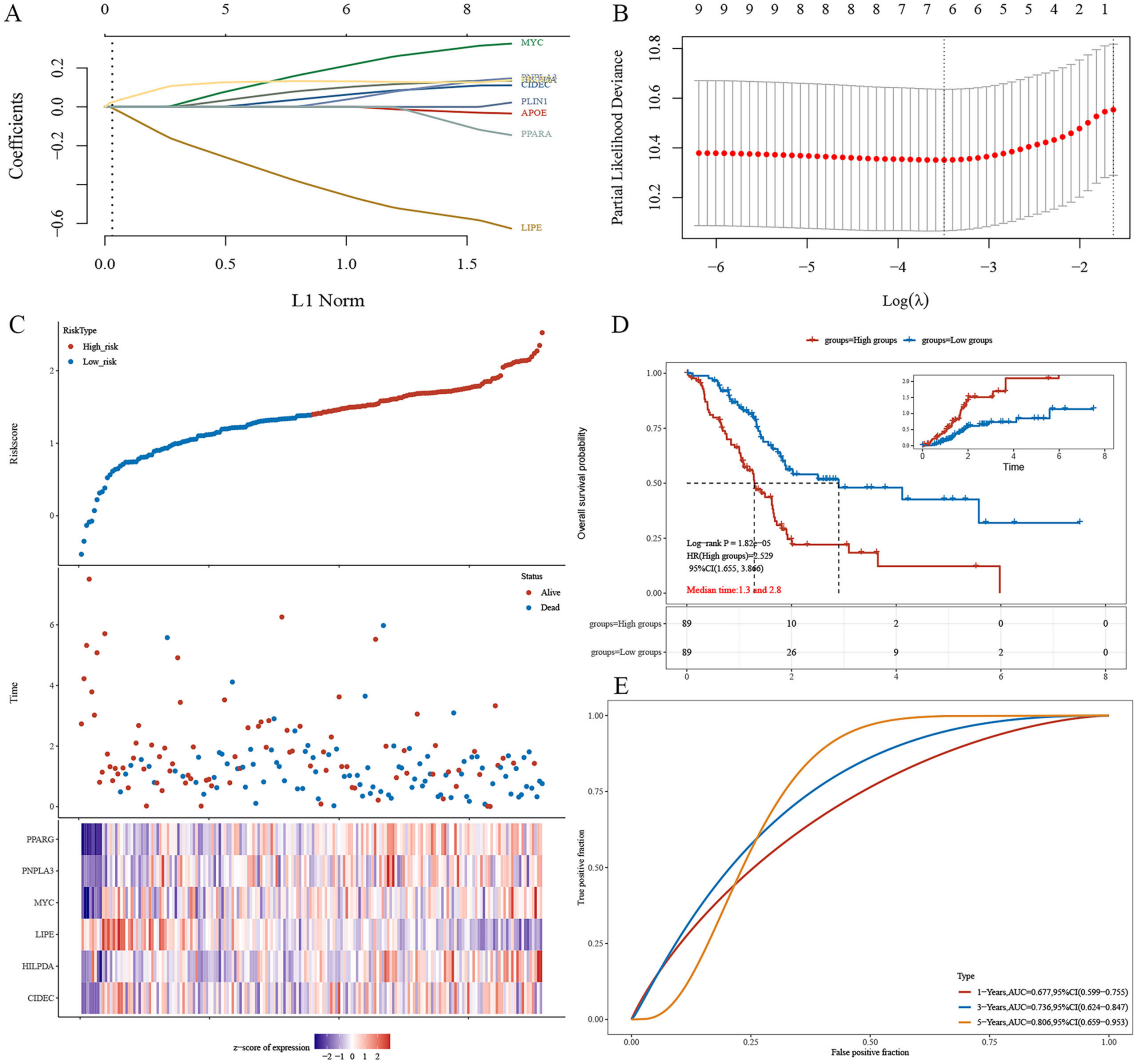
Development of a prognostic signature based on LDRGs. **(A)** LASSO coefficient profiles of the 9 prognostic LDRGs. **(B)** Confidence intervals with different values of lambda. **(C)** Patterns of risk score, patient survival status, and the expression levels of the six prognostic LDRGs. **(D)** OS curve of PAAD patients in the high-/low-expression group. **(E)** 1-, 3-, and 5-year ROC prediction curves for PAAD patients.

Survival-associated genes were identified through univariate Cox regression analysis, revealing nine genes possessing prognostic significance: APOE, CIDEC, HILPDA, LIPE, MYC, PLIN1, PNPLA3, PPARA, and PPARG (**Figure 3**). The results suggested a poorer survival rate in PAAD patients with low expression of APOE (**Figure 3A**, *P* = 0.0379), LIPE (**Figure 3D**, *P* = 0.022), and PLIN1 (**Figure 3F**, *P* = 0.0498). In addition, high expression of CIDEC (**Figure 3B**, *P* = 0.0215), HILPDA (**Figure 3C**, *P* = 0.00449), MYC (**Figure 3E**, *P* = 0.00985), PNPLA3 (**Figure 3G**, *P* = 0.0164), PPARA (**Figure 3H**, *P* = 0.0247), and PPARG (**Figure 3I**, *P* = 0.012) was also associated with poorer prognosis. A prognostic model was subsequently developed using these 9 LDRGs via LASSO Cox regression (**Figures 4A, B**). Utilizing the calculated risk scores, PAAD patients were categorized into high-risk and low-risk cohorts. Those assigned to the high-risk group demonstrated a markedly worse survival probability (**Figure 4C**). The Kaplan–Meier curve suggested that individuals with PAAD who exhibited elevated risk scores demonstrated a significantly reduced probability of overall survival compared to those with lower scores (**Figure 4D**, *P* < 0.001, HR = 2.529, 95% CI = 1.655–3.866). The area under the curve (AUC) values for the receiver operating characteristic (ROC) analyses at one, three, and five years were calculated as 0.677 (95% CI = 0.599-0.755), 0.736 (95% CI = 0.624-0.847) and 0.806 (95% CI = 0.659-0.953), respectively, as illustrated in **Figure 4E**.

### Construction and evaluation of a predictive nomogram in PAAD

**Figure 5:**
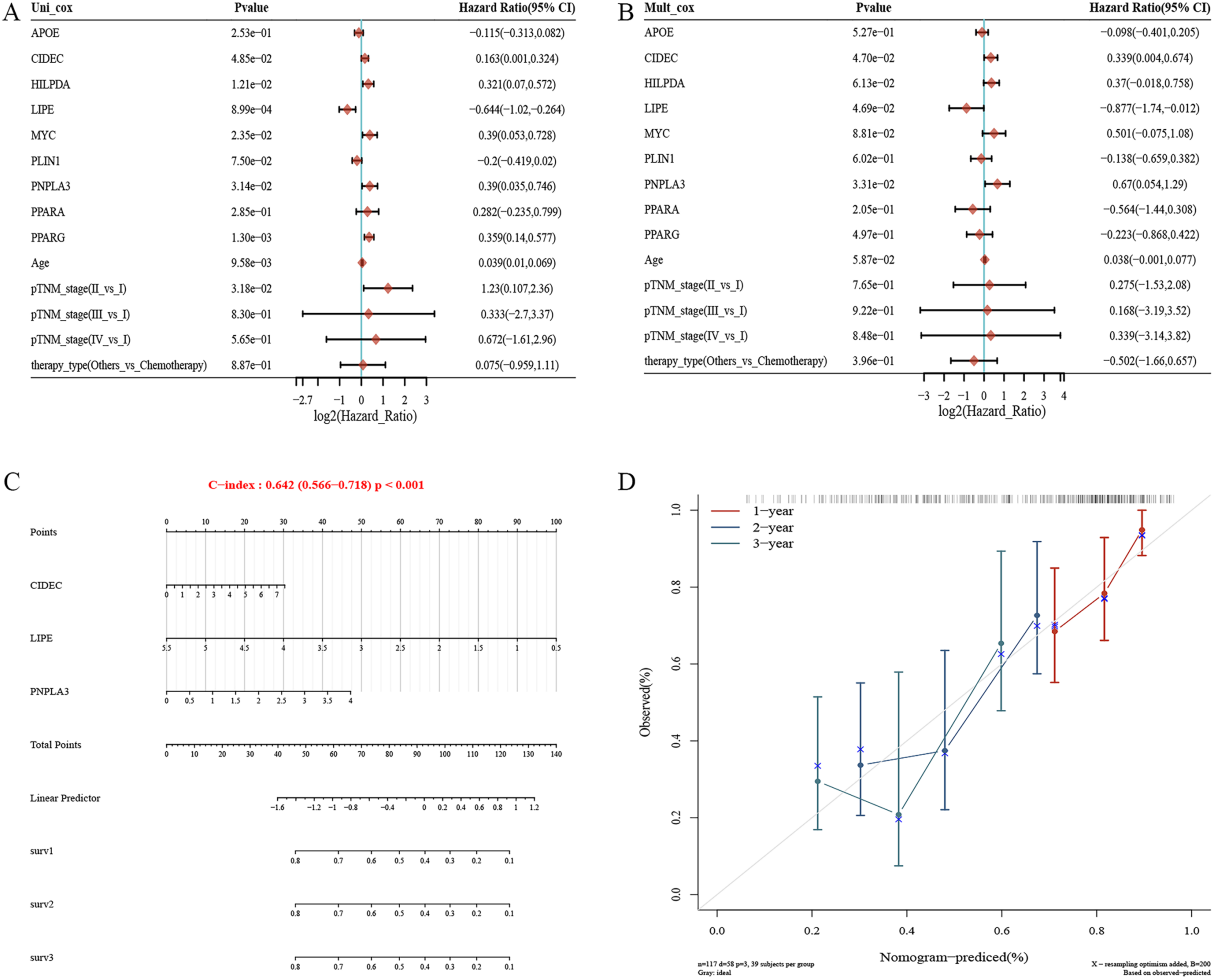
Nomogram to estimate survival probability of PAAD patients. **(A, B)** Evaluation of clinical variables and 9 prognostic LDRGs in PAAD using univariate and multivariate Cox regression. **(C)** The nomogram was designed to estimate overall survival at 1, 2, and 3 years for PAAD patients. **(D)** Calibration plots for assessing the predictive performance of the nomogram.

By integrating these prognostic LDRGs along with clinicopathological features, a predictive nomogram was formulated to assess survival likelihood. Both univariate and multivariate regression analyses revealed that CIDEC, LIPE, and PNPLA3 served as independent determinants influencing the outcomes for individuals with PAAD (**Figures 5A, B**). Subsequently, a clinical prediction model in the form of a nomogram was established, incorporating CIDEC, LIPE, and PNPLA3. The nomogram demonstrated that predictions for 1-year, 2-year, and 3-year survival probabilities were sufficiently accurate when compared with an ideal reference model (**Figures 5C, D**).

### Prognostic LDRGs interfere with tumor immune processes in PAAD

**Figure 6:**
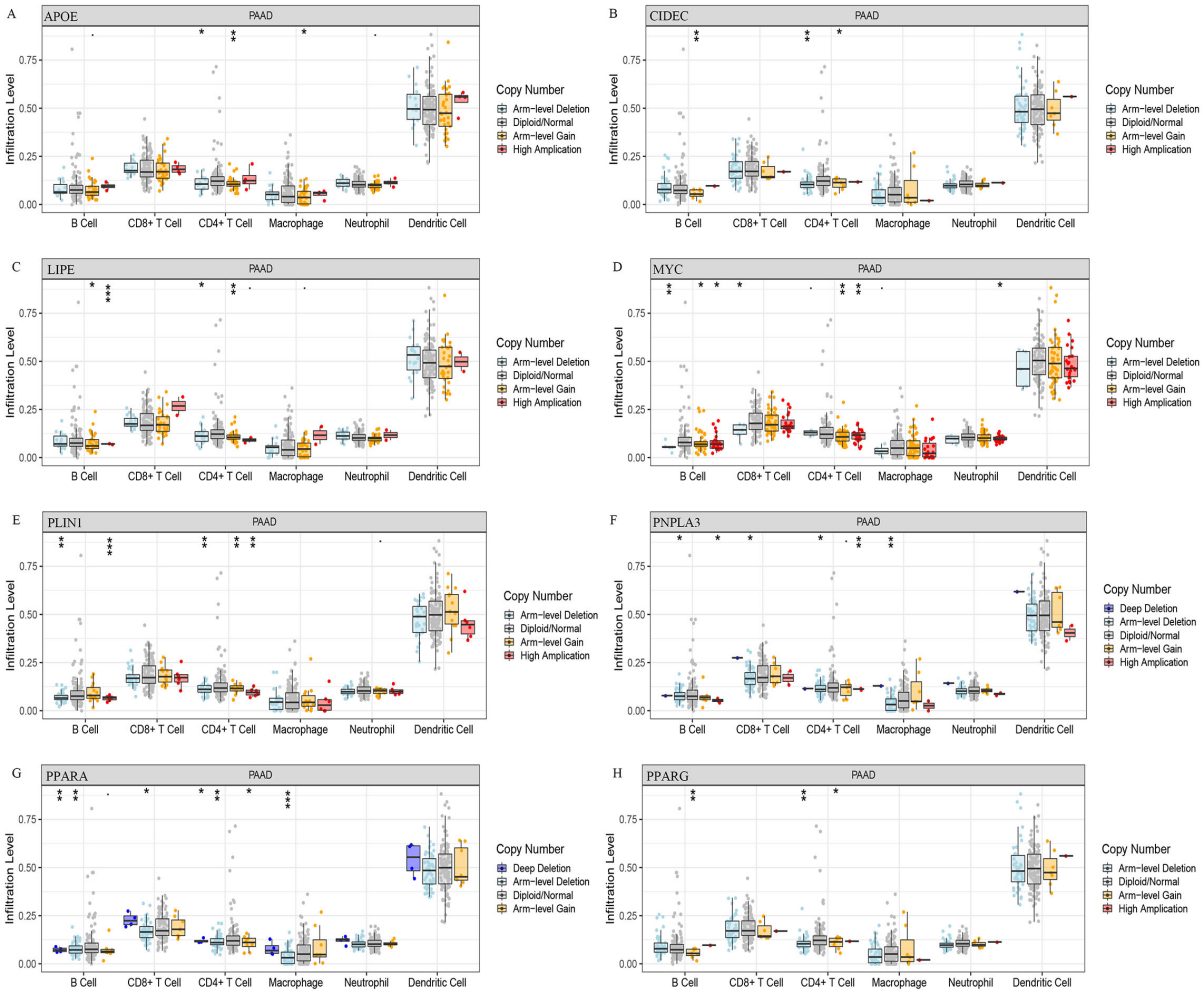
Immune cell infiltration levels in PAAD with prognostic LDRGs of varying copy numbers. **(A)** APOE **(B)** CIDEC **(C)** LIPE **(D)** MYC **(E)** PLIN1 **(F)** PNPLA3 **(G)** PPARA **(H)** PPARG *P < 0.05, **P < 0.01, ***P < 0.001.

The correlation between prognostic LDRGs and the levels of immune-infiltrating cells in PAAD was analyzed. The data revealed that APOE (**Supplementary Figure 1A**) was significantly associated with CD4+ T cells (r = 0.446, *P* = 1.17e-09), macrophages (r = 0.262, *P* = 5.31e-04), neutrophils (r = 0.292, *P* = 1.04e-04), and dendritic cells (r = 0.301, *P* = 6.21e-05). CIDEC (**Supplementary Figure 1B**) was significantly associated with B cells (r = 0.296, *P* = 3.65e-04), CD8+ T cells (r = 0.199, *P* = 9.08e-03), neutrophils (r = 0.201, *P* = 8.31e-03), and dendritic cells (r = 0.159, *P* = 3.80e-02). HILPDA, also referred to as C7orf68 (**Supplementary Figure 1C**), demonstrated significant correlations with CD8+ T cells (r = 0.367, *P* = 7.88e-07), CD4+ T cells (r = -0.158, *P* = 4.08e-02), macrophages (r = 0.306, *P *= 4.57e-05), neutrophils (r = 0.325, *P* = 1.42e-05), and dendritic cells (r = 0.292, *P* = 1.08e-04). LIPE (**Supplementary Figure 1D**) was significantly associated with B cells (r = 0.241, P = 1.51e-03), CD8+ T cells (r = 0.2, *P* = 8.69e-03), CD4+ T cells (r = 0.363, *P* = 1.22e-06), macrophages (r = 0.209, *P* = 6.20e-03), neutrophils (r = 0.163, *P* = 3.30e-02), and dendritic cells (r = 0.212, *P* = 5.3e-03). MYC (**Supplementary Figure 1E**) expression demonstrated statistically significant associations across multiple immune cell types, including B cells (r = 0.388, *P* = 1.53e-07), CD8+ T cells (r = 0.343, *P* = 4.33e-06), CD4+ T cells (r = 0.193, *P* = 1.18e-02), macrophages (r = 0.311, *P* = 3.44e-05), neutrophils (r = 0.269, *P* = 3.80e-04), and dendritic cells (r = 0.313, *P* = 3.16e-05). The analysis of PLIN1 (**Supplementary Figure 2A**) revealed significant correlations with B cells (r = 0.22, *P* = 3.89e-03), CD8+ T cells (r = 0.151, *P* = 4.80e-02), CD4+ T cells (r = 0.398, *P* = 8.53e-08), macrophages (r = 0.188, *P* = 1.40e-02), and neutrophils (r = 0.197, *P* = 9.67e-03). PPARA (**Supplementary Figure 2C**) was significantly associated with B cells (r = 0.364, *P* = 1.01e-06), CD8+ T cells (r = 0.512, *P* = 8.38e-13), macrophages (r = 0.438, *P* = 2.04e-09), neutrophils (r = 0.248, *P* = 1.06e-03), and dendritic cells (r = 0.316, *P* = 2.60e-05). In contrast, the expression levels of PNPLA3 and PPARG did not exhibit any significant correlation with immune-infiltrating cells (**Supplementary Figures 2B, D**). These prognostic LDRGs showed positive correlations with a range of immunoinhibitors and immunostimulators, with the exception of PPARG (**Supplementary Figure 2E**). Moreover, we also showed that somatic copy number alterations in APOE (**Figure 6A**), CIDEC (**Figure 6B**), LIPE (**Figure 6C**), MYC (**Figure 6D**), PLIN1 (**Figure 6E**), PNPLA3 (**Figure 6F**), PPARA (**Figure 6G**), and PPARG (**Figure 6H**) were capable of suppressing immune cell infiltration to some extent, while HILPDA was not detected.

### Construction of a regulatory network of CeRNA (LncRNA–miRNA–mRNA)

**Figure 7:**
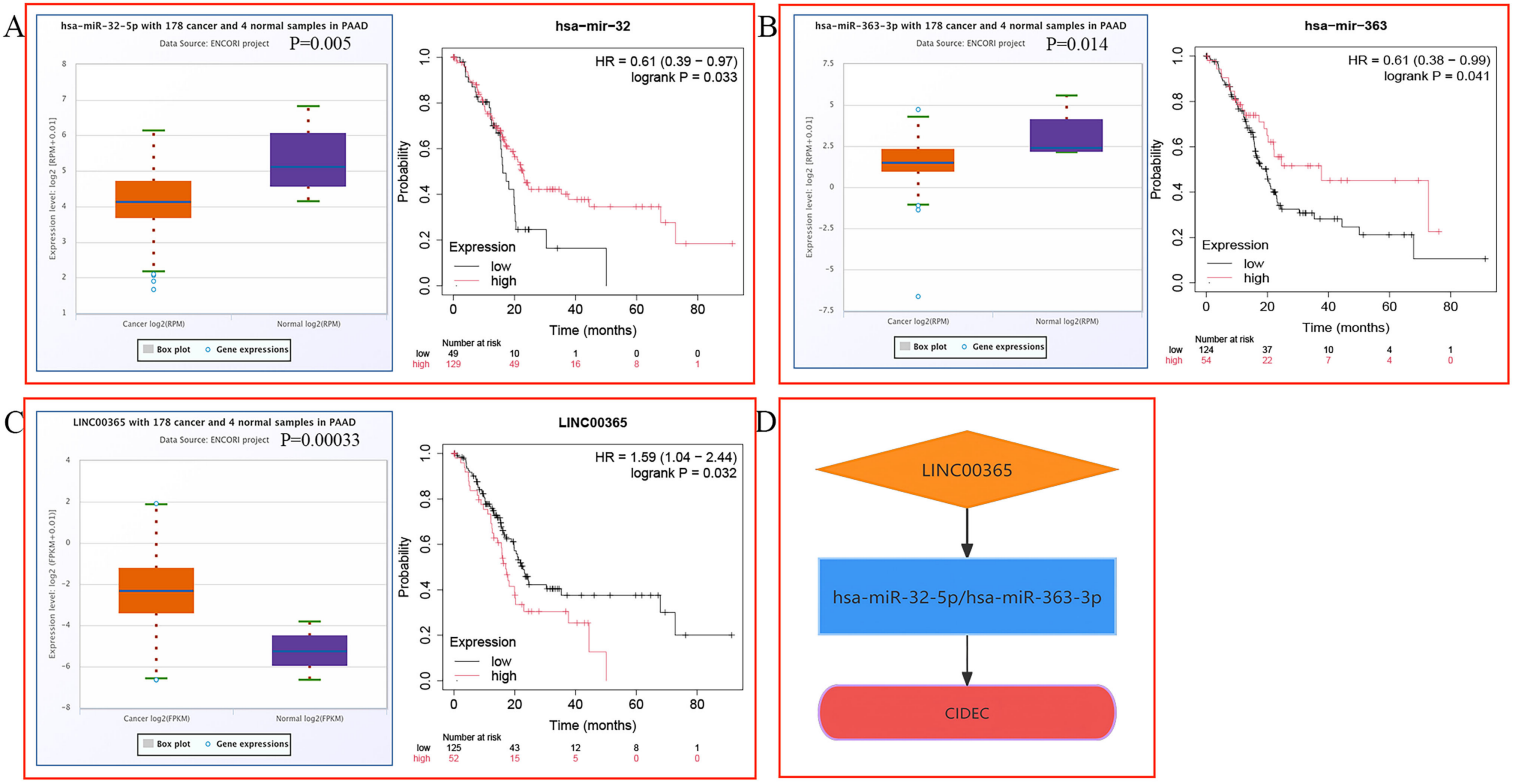
Construction of the ceRNA regulatory network in PAAD. **(A-C)** Kaplan-Meier survival curves and expression profiles for hsa-miR-32-5p **(A)**, hsa-miR-363-3p **(B)**, LINC00365 **(C)** in PAAD. **(D)** Schematic diagram of the ceRNA regulatory network.

Regression and survival analyses suggested that CIDEC might be an independent biomarker in PAAD. Consequently, CIDEC was chosen for subsequent investigation to thoroughly examine the potential regulatory role of ceRNA network in PAAD. According to the ceRNA theory, lncRNAs can function as negative regulators for miRNAs and positive regulators for mRNAs (19). Utilizing the starbase database, we inversely identified 7 potential miRNA targets of CIDEC (**Supplementary Table 2**) and assessed their expression levels along with prognostic significance in PAAD. The findings revealed that down-regulation of hsa-miR-32-5p (**Figure 7A**, *P* = 0.005) and hsa-miR-363-3p (**Figure 7B**, *P* = 0.014) was related to worse prognosis in PAAD patients (**Figure 7A**, *P* = 0.033, **Figure 7B**, *P* = 0.041). Furthermore, we investigated the upstream lncRNAs for hsa-miR-32-5p and hsa-miR-363-3p via the miRNet database, uncovering 82 miRNA-lncRNA interaction pairs (**Supplementary Table 3**). The results demonstrated that only the up-regulation of LINC00365 (an upstream lncRNA for both hsa-miR-32-5p and hsa-miR-363-3p) (**Figure 7C**, *P* = 0.00033) was correlated with poor prognosis in PAAD patients (**Figure 7C**, *P* = 0.032). To conclude the analysis, a triple regulatory network involving LncRNA, miRNA, and mRNA, linked to the advancement of PAAD, was constructed. This network incorporated 1 mRNA (CIDEC), 2 miRNAs (hsa-miR-32-5p and hsa-miR-363-3p), and 1 lncRNA (LINC00365) (**Figure 7D**).

### CIDEC affects the biological behaviors of PAAD cells *in vitro*

**Figure 8:**
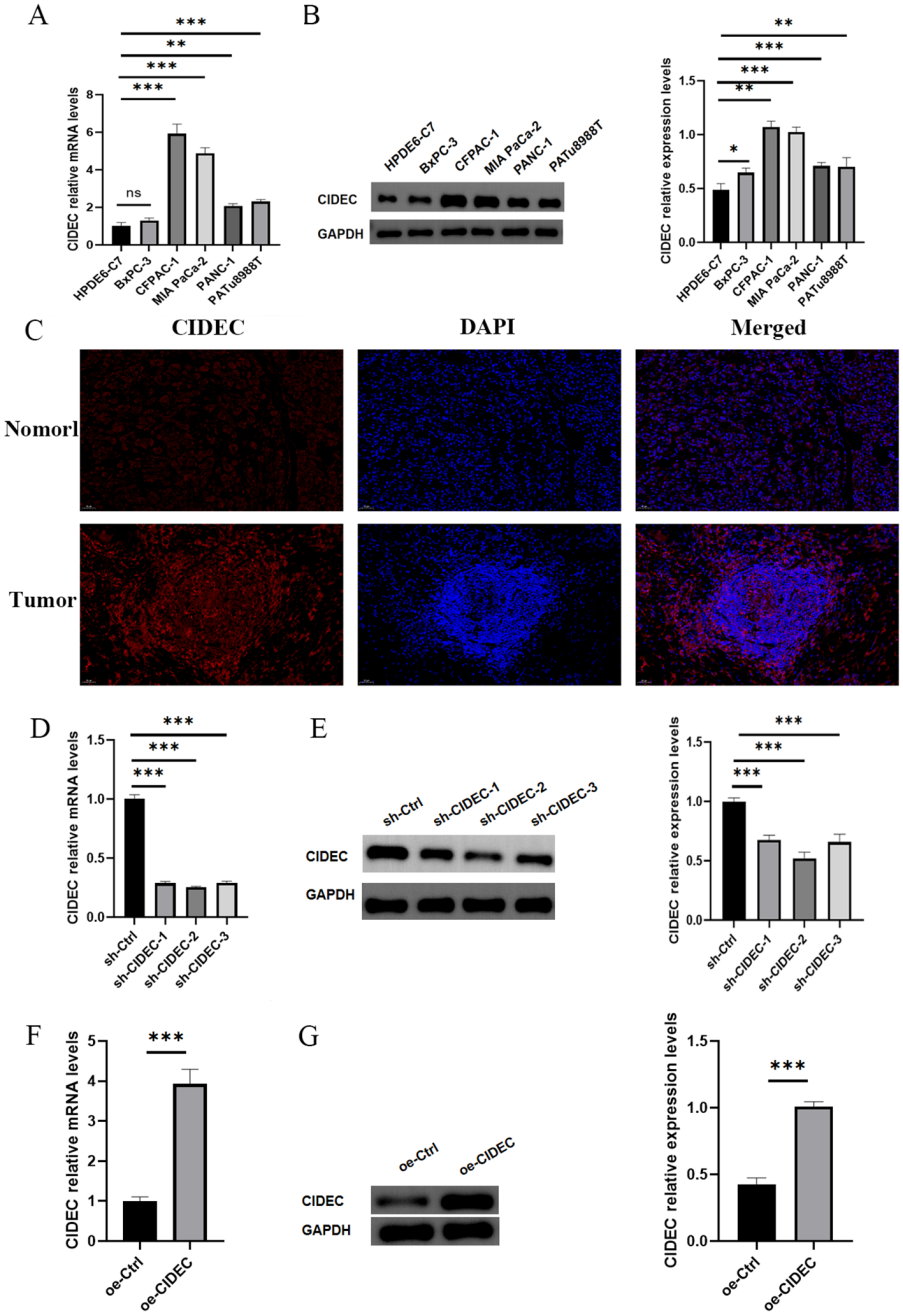
CIDEC expression levels in PAAD. **(A)** QPCR was performed to assess the differential expression of CIDEC between HPDE6-C7 and a panel of PAAD cell lines, including BxPC-3, CFPAC-1, MIA PaCa-2, PANC-1, and PATu8988T. **(B)** The expression of CIDEC at the western blot level. **(C)** Representative immunofluorescence images showing CIDEC (red), DAPI (blue), and merged signals in normal pancreas and PAAD tissues. **(D)** Assessment of CIDEC knockdown efficiency by qPCR. **(E)** Verification of CIDEC knockdown efficiency by western blotting. **(F)** CIDEC overexpression efficiency assessed by qPCR. **(G)** CIDEC overexpression efficiency confirmed by western blotting *P < 0.05, **P < 0.01, ***P < 0.001.

**Figure 9:**
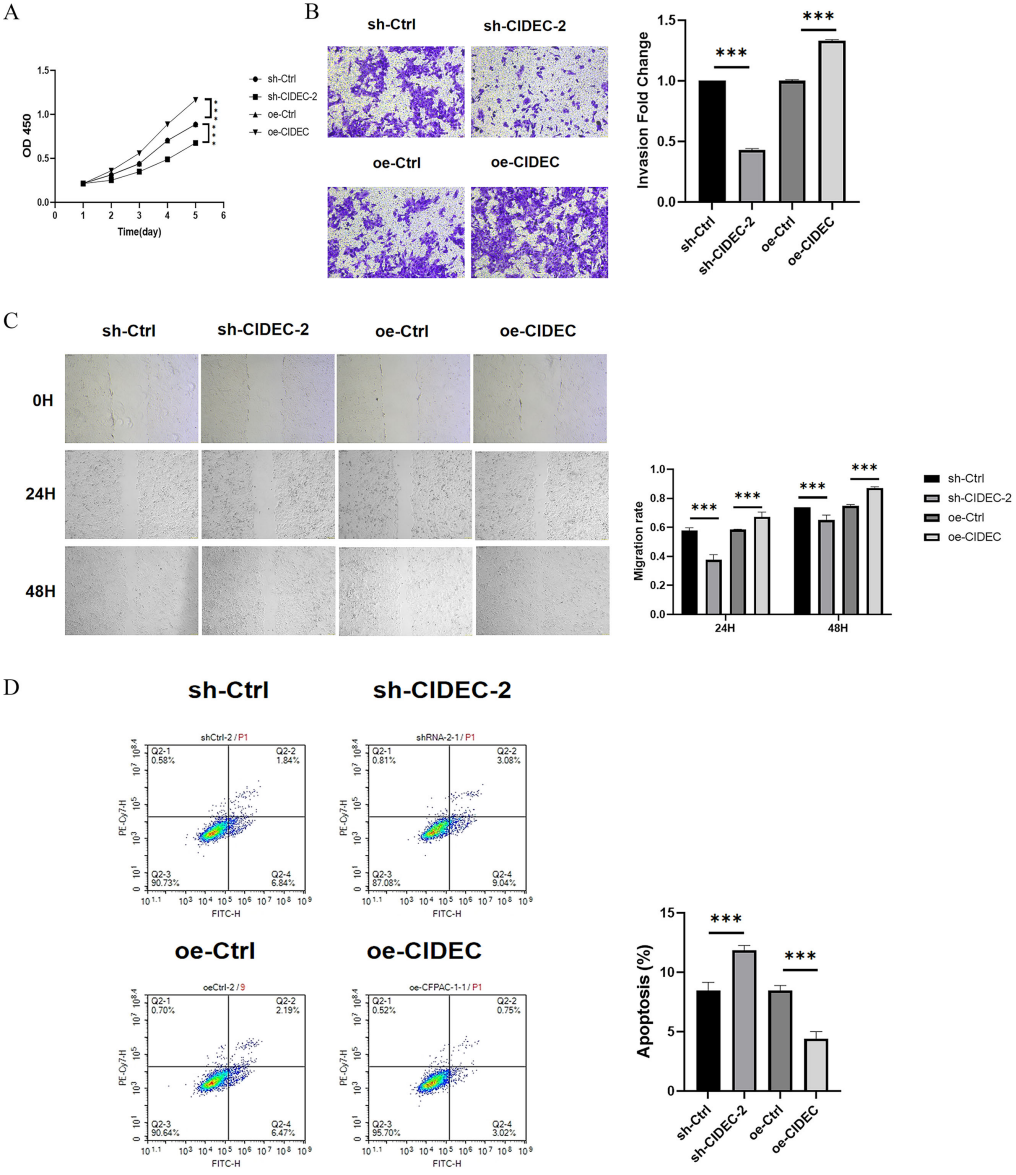
The influence of CIDEC on proliferation, migration, invasion, and apoptosis in CFPAC-1 cells. **(A)** CCK-8 assay measured the proliferative ability of CFPAC-1 cells. **(B)** Transwell assay was performed to evaluate CFPAC-1 cell invasive ability. **(C)** Wound healing assay identified the migratory ability of CFPAC-1 cells. **(D)** Flow cytometry detected apoptosis in CFPAC-1 cells. ***P < 0.001.

The expression levels of CIDEC in both a normal human pancreatic duct epithelial cell line (HPDE6-C7) and a panel of PAAD cell lines (BxPC-3, CFPAC-1, MIA PaCa-2, PANC-1, and PATu8988T) were assessed using qPCR and Western blotting. According to qPCR data, the relative abundance of CIDEC mRNA was notably elevated in the CFPAC-1, MIA PaCa-2, PANC-1, and PATu8988T cell lines compared to the HPDE6-C7 control cells (**Figure 8A**). Western blot analysis further confirmed that CIDEC protein levels were substantially increased in all tested PAAD cell lines (BxPC-3, CFPAC-1, MIA PaCa-2, PANC-1, and PATu8988T) relative to the normal HPDE6-C7 cells (**Figure 8B**). At the tissue level, immunofluorescence showed that CIDEC expression was weak and diffuse in normal pancreas but markedly upregulated in PAAD tissues, displaying a granular cytoplasmic localization with no colocalization with DAPI-stained nuclei (**Figure 8C**). We ultimately selected CFPAC-1 cells for CIDEC knockdown and overexpression experiments. The efficiency of transfection was validated by Western blotting and qPCR analysis. In CFPAC-1 cells, following transfection with sh-CIDEC-1, sh-CIDEC-2, and sh-CIDEC-3, both the mRNA and protein levels of CIDEC were significantly reduced, with sh-CIDEC-2 showing the most pronounced effect(**Figures 8D, E**). Conversely, CIDEC overexpression increased CIDEC mRNA and protein levels in CFPAC-1 cells (**Figures 8F, G**). Following this, we conducted functional studies on CFPAC-1 cells. The CCK-8 assay showed that the oe-CIDEC group exhibited significantly greater proliferation capacity than the oe-Ctrl group (**Figure 9A**). Transwell assays revealed that the oe-CIDEC group significantly promoted the invasion ability compared with oe-Ctrl group (**Figure 9B**). The wound healing assay demonstrated that the oe-CIDEC group significantly promoted the migration ability compared with oe-Ctrl group (**Figure 9C**). Moreover, apoptosis in CFPAC-1 cells was assessed using flow cytometry. The rate of apoptosis was markedly lower in the oe-CIDEC group relative to the oe-Ctrl group (**Figure 9D**). Conversely, when compared to the sh-Ctrl group, the sh-CIDEC-2 group exhibited a substantial inhibition of cell proliferation, migration, and invasion abilities, along with a significant elevation in the apoptosis rate (**Figures 9A–D**).

## Discussion

Metabolic reprogramming is a key characteristic of cancer, referring to the ability of tumor cells to reprogram metabolic pathways in response to hypoxia and nutrient deprivation, thereby accelerating cell growth and proliferation (20). Lipid droplets store and release lipids, providing raw materials for membrane synthesis and helping maintain energy homeostasis, which makes them essential for metabolic reprogramming in cancer (21). Lipid metabolic reprogramming has been reported to be closely associated between glioma, clear cell renal cell carcinoma, and intestinal tumor cells, which is thought to be involved in and regulate energy homeostasis and signaling pathways in both intracellular and extracellular spaces (22–24). It has been reported that accumulation of lipid droplets promotes treatment resistance and malignant phenotypes in PAAD (14). Several clinical outcomes in tumor patients have been associated with metabolic status, and metabolism-related risk profiles can be used as prognostic factors for cancer (25). Therefore, a predictive model integrating lipid droplet-related genomic data and clinicopathologic features offers promise for effectively predicting the prognosis of PAAD patients.

In this research, we examined the expression levels and prognostic significance of 26 LDRGs in PAAD. We found that the expression of ABHD5, APOE, AUP1, CAV1, CIDEC, CREB1, HIF1A, HILPDA, HMGCR, JUN, LDAH, LIPA, LIPE, LPIN1, MTOR, MYC, PLIN2, PLIN3, PNPLA3, PPARA, and SCD was increased in PAAD tissues relative to normal samples. In contrast, the expression of APOA5 and PLIN1 was found to be reduced. These findings are consistent with existing literature. Earlier work demonstrated that CAV1 is abundantly expressed in PAAD cells, and its suppression diminishes the cellular reserve capacity to counteract oxidative stress (26). SCD was upregulated in PAAD tissues and cells, and it might be related to the pathogenesis of the tumor (27). GO and KEGG analyses revealed that 26 LDRGs were enriched in lipid droplet, lipid localization, kaposi sarcoma−associated herpesvirus infection, regulation of lipolysis in adipocytes, cAMP signaling pathway. Interestingly, these pathways were linked to both PAAD development and treatment. The regulation of lipolysis in adipocytes pathway promotes PAAD progression through KRAS-dependent HSL activation and represents a metabolic therapeutic target (28). After activation by GPRC5A, the cAMP-CREB axis upregulates YAP1 expression at the transcriptional level, thereby accelerating PAAD progression (29).

We then performed survival analysis on 26 LDRGs and used LASSO Cox regression to develop a prognostic model from the 9 significant LDRGs: APOE, CIDEC, HILPDA, LIPE, MYC, PLIN1, PNPLA3, PPARA, and PPARG. This model looks promising for predicting overall survival in PAAD patients. Several prognostic models have already been reported for PAAD. For example, one study found a lactylation-related signature that worked well for estimating patient prognosis (30). Another study created an aging-associated genes signature and confirmed its value, proposing it as a potential therapeutic target for PAAD (31). A prognostic model integrating hypoxia and stemness traits also effectively predicted survival in PAAD patients (32). In this study, we first established a new 6 LDRGs signature (CIDEC, HILPDA, LIPE, MYC, PNPLA3, and PPARG), which provides more options for prognostic prediction in PAAD patients.

Dynamic alterations in the tumor microenvironment (TME) are not only key drivers of carcinogenesis but also critical targets for therapeutic interventions (33). Research indicates that T-cell senescence links to lipid metabolic control in the tumor microenvironment, and reshaping T-cell lipid metabolism could enhance anti-tumor immunity (34). In immune infiltration analysis, most of the prognostic LDRGs positively correlated with the infiltration levels of certain immune cells. This is similar to previous studies in other cancers. In esophageal squamous cell carcinoma, APOE may serve as a novel target for immunotherapy by reshaping the TME, with its expression linked to the infiltration of CD8+ T cells, CD4+ T cells, and dendritic cells (35). CIDEC may enhance the invasiveness of gastric adenocarcinoma by influencing the infiltration of CD8+ T cells in the TME (36).

In our study, CIDEC is one of the most important model genes. Belonging to the cell death-inducing DFF45-like effector protein family, CIDEC contributes to the regulation of multiple facets of lipid balance and homeostasis (37). However, studies of CIDEC in cancer cells are quite limited. Research indicates that suppressing CIDEC enhances the migratory and invasive capacities of lung adenocarcinoma cells (38). Through metabolic reprogramming of lipid droplets, CIDEC holds a significant position in the development of non-small cell lung cancer (39). To date, the function of CIDEC in PAAD cells has not been documented in earlier literature. In this work, we conducted experimental assays to confirm the influence of CIDEC on PAAD cells functionality, thereby supporting the credibility of our bioinformatics findings. Additionally, we established a ceRNA regulatory network centered on CIDEC and uncovered the LINC00365/hsa-miR-32-5p/hsa-miR-363-3p/CIDEC regulatory axis. A previous study showed that LINC00365 is upregulated in colorectal cancer, and its knockdown suppresses cancer cell migration and invasion, as previously demonstrated (40). In various human malignancies, such as cervical carcinoma, clear cell renal cell carcinoma, and triple-negative breast cancer, miR-32-5p has been recognized as a factor that suppresses tumor growth (41–43). Previous studies have confirmed that miR-363-3p is aberrantly expressed in colorectal cancer (44) and papillary thyroid cancer (45), and has been identified as an oncogene. The regulatory axis has not been studied before, which provided another theoretical basis for the further study of the mechanisms of PAAD progression.

CIDEC functions as a protein associated with lipid droplets, positioning itself on the surfaces of these droplets as well as on the endoplasmic reticulum; it lacks a classical signal peptide and is not actively secreted into the extracellular space (37). Therefore, CIDEC should be regarded as a tissue-based prognostic biomarker rather than a blood-secreted marker. Its expression level can be conveniently evaluated in formalin-fixed paraffin-embedded (FFPE) or fresh frozen tumor tissues using routine techniques such as immunohistochemistry (IHC) or quantitative real-time PCR (qPCR).

For clinical application, CIDEC can be measured in surgical resection or core needle biopsy samples, helping with prognosis and treatment choices. Liquid biopsy for CIDEC (e.g., from circulating tumor cells or exosomes) is theoretically possible but lacks validated assays and remains investigational. Compared with the classical serum biomarker CA19-9, CIDEC offers several complementary advantages: (i) it is applicable to all PAAD patients, including those with Lewis antigen−negative phenotype (5%–34% of pancreatic cancer patients) who produce little to no CA19-9 (46); (ii) its tissue-based measurement is not confounded by benign conditions such as biliary obstruction or cholangitis; and (iii) as a lipid droplet−resident protein, CIDEC directly participates in malignant phenotypes (proliferation, migration, invasion, and anti-apoptosis), thereby providing a more direct biological link to tumor aggressiveness. Thus, the prospective integration of CA19–9 for dynamic monitoring with CIDEC tissue−based risk stratification may enable a more refined and individualized management strategy for PAAD patients.

However, this study does have some limitations worth noting. First, although our cellular experiments have clearly demonstrated that CIDEC promotes the proliferation, migration, and invasion of PAAD cells and inhibits their apoptosis, these experiments have not elucidated the specific molecular mechanisms by which CIDEC exerts these effects. The identification of probable downstream signaling cascades, such as those involving PI3K/AKT, MAPK, or AMPK, as well as direct protein interactors of CIDEC, represents an area for future investigation. Second, although our bioinformatic analysis suggested associations between prognostic LDRGs and immune infiltration (e.g., correlations with CD8^+^ T cells, macrophages, and dendritic cells), the current *in vitro* validation did not include any immunological assays, such as co-culture systems with immune cells, assessment of immune checkpoint molecule expression upon CIDEC modulation, or *in vivo* tumor models to evaluate immune landscape changes. Despite these limitations, our study provides the first evidence linking CIDEC to PAAD aggressiveness and establishes a foundation for further mechanistic and immunological investigations.

## Conclusion

The current study identified a lipid droplet-related prognostic signature including these 6 prognostic biomarkers (CIDEC, HILPDA, LIPE, MYC, PNPLA3, and PPARG) for PAAD. In addition, we first identified the LINC00365/hsa-miR-32-5p/hsa-miR-363-3p/CIDEC regulatory axis that regulates the PAAD process. More importantly, *in vitro* studies demonstrated that elevated CIDEC expression enhances the proliferative, migratory, and invasive capabilities of PAAD cells while concurrently suppressing apoptotic processes. These findings provide initial validation that CIDEC holds potential as both a prognostic indicator and a therapeutic target for PAAD.

## Data Availability

The original contributions presented in the study are included in the article/**Supplementary Material**. Further inquiries can be directed to the corresponding authors.

## Supplementary material

Supplementary Figure 1 Association between prognostic LDRGs and immune-infiltrating cells in PAAD. A APOE B CIDEC C HILPDA D LIPE E MYC.

Supplementary Figure 2 Relationship between prognostic LDRGs and immune-infiltrating cells/immune checkpoints in PAAD. A-D The correlation of PLIN1 (A), PNPLA3 (B), PPARA (C) and PPARG (D) expression levels with immune-infiltrating cells. E The relationship between prognostic LDRGs and immune checkpoints.

Supplementary Table 1 –26 lipid droplet-related genes. displays the mRNA-miRNA pairs forecasted by the starbase database.

Supplementary Table 2 StarBase-predicted mRNA-miRNA pairs.

Supplementary Table 3 MiRNet -predicted miRNA-lncRNA pairs.

## References

[1] Rahib L Coffin T Kenner B . Factors driving pancreatic cancer survival rates. Pancreas. (2025) 54:e530–6. doi: 10.1097/mpa.0000000000002489 40245290 PMC12175802

[2] Wenzel P Mogler C Görgülü K Algül H . Pancreatic cancer: Current concepts, trends, and future directions. Turk J Gastroenterol. (2024) 36:69–81. doi: 10.5152/tjg.2024.24544 39632706 PMC11843265

[3] Bray F Laversanne M Sung H Ferlay J Siegel RL Soerjomataram I . Global cancer statistics 2022: GLOBOCAN estimates of incidence and mortality worldwide for 36 cancers in 185 countries. CA Cancer J Clin. (2024) 74:229–63. doi: 10.3322/caac.21834 38572751

[4] Strobel O Lorenz P Hinz U Gaida M König AK Hank T . Actual five-year survival after upfront resection for pancreatic ductal adenocarcinoma: Who beats the odds? Ann Surg. (2022) 275:962–71. doi: 10.1097/sla.0000000000004147 32649469

[5] Stoffel EM Brand RE Goggins M . Pancreatic cancer: Changing epidemiology and new approaches to risk assessment, early detection, and prevention. Gastroenterology. (2023) 164:752–65. doi: 10.1053/j.gastro.2023.02.012 36804602 PMC10243302

[6] Du X Zhou L Aw YC Mak HY Xu Y Rae J . ORP5 localizes to ER-lipid droplet contacts and regulates the level of PI(4)P on lipid droplets. J Cell Biol. (2020) 219:e201905162. doi: 10.1083/jcb.201905162 31653673 PMC7039201

[7] Zadoorian A Du X Yang H . Lipid droplet biogenesis and functions in health and disease. Nat Rev Endocrinol. (2023) 19:443–59. doi: 10.1038/s41574-023-00845-0 37221402 PMC10204695

[8] Zhou L Lu Y Qiu X Chen Z Tang Y Meng Z . Lipid droplet efferocytosis attenuates proinflammatory signaling in macrophages via TREM2- and MS4A7-dependent mechanisms. Cell Rep. (2025) 44:115310. doi: 10.1016/j.celrep.2025.115310 39954254 PMC11973828

[9] Kumari RM Khatri A Chaudhary R Choudhary V . Concept of lipid droplet biogenesis. Eur J Cell Biol. (2023) 102:151362. doi: 10.1016/j.ejcb.2023.151362 37742390 PMC7615795

[10] Cui P Li X Huang C Lin D . Metabolomics-driven discovery of therapeutic targets for cancer cachexia. J Cachexia Sarcopenia Muscle. (2024) 15:781–93. doi: 10.1002/jcsm.13465 38644205 PMC11154780

[11] Wang C Shi M Ji J Cai Q Zhao Q Jiang J . Stearoyl-CoA desaturase 1 (SCD1) facilitates the growth and anti-ferroptosis of gastric cancer cells and predicts poor prognosis of gastric cancer. Aging (Albany NY). (2020) 12:15374–91. doi: 10.18632/aging.103598 32726752 PMC7467382

[12] Jiang C Liu Y Wen S Xu C Gu L . In silico development and clinical validation of novel 8 gene signature based on lipid metabolism related genes in colon adenocarcinoma. Pharmacol Res. (2021) 169:105644. doi: 10.1016/j.phrs.2021.105644 33940186

[13] Tomacha J Dokduang H Padthaisong S Namwat N Klanrit P Phetcharaburanin J . Targeting fatty acid synthase modulates metabolic pathways and inhibits cholangiocarcinoma cell progression. Front Pharmacol. (2021) 12:696961. doi: 10.3389/fphar.2021.696961 34421595 PMC8371458

[14] Lin Y Zhang X Wang Y Yao W . LPCAT2-mediated lipid droplet production supports pancreatic cancer chemoresistance and cell motility. Int Immunopharmacol. (2024) 139:112681. doi: 10.1016/j.intimp.2024.112681 39068758

[15] Schwartsburd P . Lipid droplets: Could they be involved in cancer growth and cancer-microenvironment communications? Cancer Commun (Lond). (2022) 42:83–7. doi: 10.1002/cac2.12257 35029069 PMC8822591

[16] Zhao D Wu L Li Y . Targeting lipid metabolism to enhance cancer immunotherapy. Biochim Biophys Acta Rev Cancer. (2025) 1880:189416. doi: 10.1016/j.bbcan.2025.189416 40803628

[17] Aria H Azizi M Nazem S Mansoori B Darbeheshti F Niazmand A . Competing endogenous RNAs regulatory crosstalk networks: The messages from the RNA world to signaling pathways directing cancer stem cell development. Heliyon. (2024) 10:e35208. doi: 10.1016/j.heliyon.2024.e35208 39170516 PMC11337742

[18] Weng W Zhang Z Huang W Xu X Wu B Ye T . Identification of a competing endogenous RNA network associated with prognosis of pancreatic adenocarcinoma. Cancer Cell Int. (2020) 20:231. doi: 10.1186/s12935-020-01243-6 32536819 PMC7288603

[19] Yang X Li F Ma J Liu Y Wang X Wang R . Study on the relationship between the miRNA-centered ceRNA regulatory network and fatigue. J Mol Neurosci. (2021) 71:1967–74. doi: 10.1007/s12031-021-01845-3 33993410 PMC8500871

[20] Hanahan D . Hallmarks of cancer: New dimensions. Cancer Discov. (2022) 12:31–46. doi: 10.1158/2159-8290.cd-21-1059 35022204

[21] Petan T . Lipid droplets in cancer. Rev Physiol Biochem Pharmacol. (2023) 185:53–86. doi: 10.1007/112_2020_51 33074407

[22] Masui K Cavenee WK Mischel PS Shibata N . The metabolomic landscape plays a critical role in glioma oncogenesis. Cancer Sci. (2022) 113:1555–63. doi: 10.1111/cas.15325 35271755 PMC9128185

[23] Chen C Zhao W Lu X Ma Y Zhang P Wang Z . AUP1 regulates lipid metabolism and induces lipid accumulation to accelerate the progression of renal clear cell carcinoma. Cancer Sci. (2022) 113:2600–15. doi: 10.1111/cas.15445 PMC9357643 35633317

[24] Ko CW Qu J Black DD Tso P . Regulation of intestinal lipid metabolism: Current concepts and relevance to disease. Nat Rev Gastroenterol Hepatol. (2020) 17:169–83. doi: 10.1038/s41575-019-0250-7 32015520

[25] Yang C Huang S Cao F Zheng Y . A lipid metabolism-related genes prognosis biomarker associated with the tumor immune microenvironment in colorectal carcinoma. BMC Cancer. (2021) 21:1182. doi: 10.21203/rs.3.rs-145179/v1 34740325 PMC8571885

[26] Liang X Tian R Li T Wang H Qin Y Qian M . Integrative insights into the role of CAV1 in ketogenic diet and ferroptosis in pancreatic cancer. Cell Death Discov. (2025) 11:139. doi: 10.1038/s41420-025-02421-z 40180904 PMC11968908

[27] Zhang X Zhao LX Cheng SQ Liu YF . Expression of stearoyl coenzyme a desaturase in neuronal cells facilitates pancreatic cancer progression. Cancer Cell Int. (2025) 25:57. doi: 10.1186/s12935-025-03682-5 39979962 PMC11844101

[28] Rozeveld CN Johnson KM Zhang L Razidlo GL . KRAS controls pancreatic cancer cell lipid metabolism and invasive potential through the lipase HSL. Cancer Res. (2020) 80:4932–45. doi: 10.1158/0008-5472.can-20-1255 32816911 PMC7669720

[29] Fang W Yu X Deng J Yu B Xiong J Ma M . Upregulated GPRC5A disrupting the Hippo pathway promotes the proliferation and migration of pancreatic cancer cells via the cAMP-CREB axis. Discov Oncol. (2023) 14:17. doi: 10.1007/s12672-023-00626-1 36735162 PMC9898488

[30] Peng T Sun F Yang JC Cai MH Huai MX Pan JX . Novel lactylation-related signature to predict prognosis for pancreatic adenocarcinoma. World J Gastroenterol. (2024) 30:2575–602. doi: 10.3748/wjg.v30.i19.2575 38817665 PMC11135411

[31] Xue S Ge W Wang K Mao T Zhang X Xu H . Association of aging-related genes with prognosis and immune infiltration in pancreatic adenocarcinoma. Front Cell Dev Biol. (2022) 10:942225. doi: 10.3389/fcell.2022.942225 36003146 PMC9393218

[32] Tian X Zheng J Mou W Lu G Chen S Du J . Development and validation of a hypoxia-stemness-based prognostic signature in pancreatic adenocarcinoma. Front Pharmacol. (2022) 13:939542. doi: 10.3389/fphar.2022.939542 35935823 PMC9350896

[33] Bilotta MT Antignani A Fitzgerald DJ . Managing the TME to improve the efficacy of cancer therapy. Front Immunol. (2022) 13:954992. doi: 10.3389/fimmu.2022.954992 36341428 PMC9630343

[34] Liu X Hartman CL Li L Albert CJ Si F Gao A . Reprogramming lipid metabolism prevents effector T cell senescence and enhances tumor immunotherapy. Sci Transl Med. (2021) 13:eaaz6314. doi: 10.1126/scitranslmed.aaz6314 33790024 PMC12040281

[35] Zhang Z Jin G Zhao J Deng S Chen F Wuyun G . Mitochondrial energy metabolism correlates with an immunosuppressive tumor microenvironment and poor prognosis in esophageal squamous cell carcinoma. Comput Struct Biotechnol J. (2023) 21:4118–33. doi: 10.1016/j.csbj.2023.08.022 37664173 PMC10474161

[36] Pan D Chen H Xu J Lin X Li L . Evaluation of vital genes correlated with CD8 + T cell infiltration as prognostic biomarkers in stomach adenocarcinoma. BMC Gastroenterol. (2023) 23:399. doi: 10.1186/s12876-023-03003-y 37978443 PMC10656896

[37] Xu L Li L Wu L Li P Chen FJ . CIDE proteins and their regulatory mechanisms in lipid droplet fusion and growth. FEBS Lett. (2024) 598:1154–69. doi: 10.1002/1873-3468.14823 38355218

[38] Guo F Yuan D Zhang J Zhang H Wang C Zhu L . Silencing of ARL14 gene induces lung adenocarcinoma cells to a dormant state. Front Cell Dev Biol. (2019) 7:238. doi: 10.3389/fcell.2019.00238 31750299 PMC6843082

[39] Chen Y Sun Y Gong L Yi Q Liu L Zou W . Cell death-inducing DFF45-like effector C intervenes the progression of non-small cell lung cancer via modulating lipid metabolism. Int J Surg. (2025) 111(10):6688–704. doi: 10.1097/js9.0000000000002796 40552859 PMC12527760

[40] Yang W Huang X Lv W Jin Y Zhu Y . LINC00365 promotes miR-221-5p to inhibit pyroptosis via Dicer in colorectal cancer. Acta Biochim Biophys Sin (Shanghai). (2024) 57:529–41. doi: 10.3724/abbs.2024173 39439418 PMC12040748

[41] Liu YJ Zhou HG Chen LH Qu DC Wang CJ Xia ZY . MiR-32-5p regulates the proliferation and metastasis of cervical cancer cells by targeting HOXB8. Eur Rev Med Pharmacol Sci. (2019) 23:87–95. doi: 10.26355/eurrev_201901_16752 30657550

[42] Wang M Sun Y Xu J Lu J Wang K Yang DR . Preclinical studies using miR-32-5p to suppress clear cell renal cell carcinoma metastasis via altering the miR-32-5p/TR4/HGF/Met signaling. Int J Cancer. (2018) 143:100–12. doi: 10.1002/ijc.31289 29396852 PMC5938119

[43] Yang J Niu H Chen X . GATA1-activated HNF1A-AS1 facilitates the progression of triple-negative breast cancer via sponging miR-32-5p to upregulate RNF38. Cancer Manag Res. (2021) 13:1357–69. doi: 10.2147/cmar.s274204 33603481 PMC7886384

[44] Wang Y Bai SK Zhang T Liao CG . MicroRNA-363-3p inhibits colorectal cancer progression by targeting interferon-induced transmembrane protein 1. World J Gastrointest Oncol. (2023) 15:1556–66. doi: 10.4251/wjgo.v15.i9.1556 37746648 PMC10514722

[45] Dong S Xue S Sun Y Han Z Sun L Xu J . MicroRNA-363-3p downregulation in papillary thyroid cancer inhibits tumor progression by targeting NOB1. J Investig Med. (2021) 69:66–74. doi: 10.1136/jim-2020-001562 33077486 PMC7803892

[46] Luo G Liu C Guo M Cheng H Lu Y Jin K . Potential biomarkers in Lewis negative patients with pancreatic cancer. Ann Surg. (2017) 265:800–5. doi: 10.1097/sla.0000000000001741 28267695

