# Supplementary material for: A novel lipid droplet-related prognostic signature reveals CIDEC as a key biomarker for pancreatic adenocarcinoma
Source: Front Oncol. 2026 Jul 28;16:1845259. doi: 10.3389/fonc.2026.1845259 (PMC13456994; doi:10.3389/fonc.2026.1845259)
Supplement: Supplementary file 1 [file Table1.docx]

**Table S1 26 lipid droplet-related genes**

| **Genes** | **Full-names** |
| --- | --- |
| PNPLA2 | Patatin Like Phospholipase Domain Containing 2 |
| APOE | Apolipoprotein E |
| PPARG | Peroxisome Proliferator Activated Receptor Gamma |
| HILPDA | Hypoxia Inducible Lipid Droplet Associated |
| LDAH | Lipid Droplet Associated Hydrolase |
| PLIN1 | Perilipin 1 |
| PPARA | Peroxisome Proliferator Activated Receptor Alpha |
| LPIN1 | Lipin 1 |
| ABHD5 | Abhydrolase Domain Containing 5, Lysophosphatidic Acid Acyltransferase |
| MTOR | Mechanistic Target Of Rapamycin Kinase |
| APOA5 | Apolipoprotein A5 |
| HMGCR | 3-Hydroxy-3-Methylglutaryl-CoA Reductase |
| PLIN2 | Perilipin 2 |
| CIDEC | Cell Death Inducing DFFA Like Effector C |
| LIPA | Lipase A, Lysosomal Acid Type |
| CAV1 | Caveolin 1 |
| MYC | MYC Proto-Oncogene, BHLH Transcription Factor |
| JUN | Jun Proto-Oncogene, AP-1 Transcription Factor Subunit |
| LIPE | Lipase E, Hormone Sensitive Type |
| HIF1A | Hypoxia Inducible Factor 1 Subunit Alpha |
| AUP1 | AUP1 Lipid Droplet Regulating VLDL Assembly Factor |
| CREB1 | CAMP Responsive Element Binding Protein 1 |
| CIDEA | Cell Death Inducing DFFA Like Effector A |
| SCD | Stearoyl-CoA Desaturase |
| PNPLA3 | Patatin Like Phospholipase Domain Containing 3 |
| PLIN3 | Perilipin 3 |
