# Supplementary material for: A novel lipid droplet-related prognostic signature reveals CIDEC as a key biomarker for pancreatic adenocarcinoma
Source: Front Oncol. 2026 Jul 28;16:1845259. doi: 10.3389/fonc.2026.1845259 (PMC13456994; doi:10.3389/fonc.2026.1845259)
Supplement: Supplementary file 2 [file Table2.docx]

**Table S2 The mRNA-miRNA pairs predicted by starbase database**

| **mRNA** | **miRNA** |
| --- | --- |
| CIDEC | hsa-miR-25-3p |
| CIDEC | hsa-miR-32-5p |
| CIDEC | hsa-miR-92a-3p |
| CIDEC | hsa-miR-137 |
| CIDEC | hsa-miR-363-3p |
| CIDEC | hsa-miR-367-3p |
| CIDEC | hsa-miR-92b-3p |
