# Supplementary material for: A novel lipid droplet-related prognostic signature reveals CIDEC as a key biomarker for pancreatic adenocarcinoma
Source: Front Oncol. 2026 Jul 28;16:1845259. doi: 10.3389/fonc.2026.1845259 (PMC13456994; doi:10.3389/fonc.2026.1845259)
Supplement: Supplementary file 3 [file Table3.docx]

**Table S3 The miRNA-lncRNA pairs predicted by miRNet database**

| **miRNA** | **lncRNA** |
| --- | --- |
| hsa-miR-32-5p | LINC01128 |
| hsa-miR-32-5p | MDS2 |
| hsa-miR-32-5p | LINC00467 |
| hsa-miR-32-5p | PAX8-AS1 |
| hsa-miR-32-5p | WWTR1-IT1 |
| hsa-miR-32-5p | PURPL |
| hsa-miR-32-5p | NR2F1-AS1 |
| hsa-miR-32-5p | MIR3936HG |
| hsa-miR-32-5p | SNHG4 |
| hsa-miR-32-5p | SNHG5 |
| hsa-miR-32-5p | FEZF1-AS1 |
| hsa-miR-32-5p | WEE2-AS1 |
| hsa-miR-32-5p | DAPK1-IT1 |
| hsa-miR-32-5p | LINC00963 |
| hsa-miR-32-5p | LINC00858 |
| hsa-miR-32-5p | KCNQ1OT1 |
| hsa-miR-32-5p | MALAT1 |
| hsa-miR-32-5p | TBX5-AS1 |
| hsa-miR-32-5p | HNF1A-AS1 |
| hsa-miR-32-5p | LINC00365 |
| hsa-miR-32-5p | INTS6-AS1 |
| hsa-miR-32-5p | LINC02321 |
| hsa-miR-32-5p | LINC01550 |
| hsa-miR-32-5p | MEG8 |
| hsa-miR-32-5p | PWAR5 |
| hsa-miR-32-5p | SNHG14 |
| hsa-miR-32-5p | ARHGAP11B |
| hsa-miR-32-5p | OIP5-AS1 |
| hsa-miR-32-5p | PLA2G4E-AS1 |
| hsa-miR-32-5p | GABPB1-IT1 |
| hsa-miR-32-5p | GABPB1-AS1 |
| hsa-miR-32-5p | ST20-AS1 |
| hsa-miR-32-5p | PITPNA-AS1 |
| hsa-miR-32-5p | CCDC144NL-AS1 |
| hsa-miR-32-5p | LINC01915 |
| hsa-miR-32-5p | NORAD |
| hsa-miR-32-5p | SNHG17 |
| hsa-miR-32-5p | DUXAP8 |
| hsa-miR-32-5p | LINC01560 |
| hsa-miR-32-5p | XIST |
| hsa-miR-32-5p | JPX |
| hsa-miR-363-3p | LINC01128 |
| hsa-miR-363-3p | MDS2 |
| hsa-miR-363-3p | LINC00467 |
| hsa-miR-363-3p | C1orf143 |
| hsa-miR-363-3p | PAX8-AS1 |
| hsa-miR-363-3p | WWTR1-IT1 |
| hsa-miR-363-3p | PURPL |
| hsa-miR-363-3p | NR2F1-AS1 |
| hsa-miR-363-3p | MIR3936HG |
| hsa-miR-363-3p | SNHG4 |
| hsa-miR-363-3p | SNHG5 |
| hsa-miR-363-3p | FEZF1-AS1 |
| hsa-miR-363-3p | WEE2-AS1 |
| hsa-miR-363-3p | DAPK1-IT1 |
| hsa-miR-363-3p | LINC00963 |
| hsa-miR-363-3p | LINC00858 |
| hsa-miR-363-3p | KCNQ1OT1 |
| hsa-miR-363-3p | MALAT1 |
| hsa-miR-363-3p | TBX5-AS1 |
| hsa-miR-363-3p | HNF1A-AS1 |
| hsa-miR-363-3p | LINC00365 |
| hsa-miR-363-3p | INTS6-AS1 |
| hsa-miR-363-3p | LINC02321 |
| hsa-miR-363-3p | LINC01550 |
| hsa-miR-363-3p | MEG8 |
| hsa-miR-363-3p | PWAR5 |
| hsa-miR-363-3p | SNHG14 |
| hsa-miR-363-3p | ARHGAP11B |
| hsa-miR-363-3p | OIP5-AS1 |
| hsa-miR-363-3p | PLA2G4E-AS1 |
| hsa-miR-363-3p | GABPB1-IT1 |
| hsa-miR-363-3p | GABPB1-AS1 |
| hsa-miR-363-3p | PITPNA-AS1 |
| hsa-miR-363-3p | CCDC144NL-AS1 |
| hsa-miR-363-3p | LINC01915 |
| hsa-miR-363-3p | NORAD |
| hsa-miR-363-3p | SNHG17 |
| hsa-miR-363-3p | DUXAP8 |
| hsa-miR-363-3p | LINC01560 |
| hsa-miR-363-3p | XIST |
| hsa-miR-363-3p | JPX |
